# Supplementary material for: Quantifying seasonal to multi-decadal signals in coastal water quality using high- and low-frequency time series data
Source: Camb Prism Coast Futur. 2024 Apr 29;2:e8. doi: 10.1017/cft.2024.6 (PMC12337606; doi:10.1017/cft.2024.6)
Supplement: Brahmey et al. supplementary material [file S2754720524000064sup001.docx]

**Supplemental Materials**

**Quantifying seasonal to multi-decadal signals in coastal water quality using high- and low-frequency time series data**

Emma I. Brahmey, Karen J. McGlathery, Scott C. Doney

| **Table S1**. VCR-LTER and ESL sites temperature (℃) composite harmonic elements where percent variance of the first harmonic ($pV_{1}$) and the second harmonic ($pV_{2}$) are in %, minimum, maximum, and amplitude ($A_{max}$) values are in ℃ and time of minima ($t_{min}$) and maxima ($t_{max}$) are in year day. | | | | | | | | |
| --- | --- | --- | --- | --- | --- | --- | --- | --- |
| Site | $pV_{1}$ | $pV_{2}$ | $t_{min}$ | Minimum Value | $t_{max}$ | Maximum Value | $A_{max}$ | $\chi_{v}^{2}$ |
| RBCM | 91.4 | 8.6 | 19.0 ± 0.8 | 6.53 ± 0.12 | 213.4 ± 0.6 | 30.18 ± 0.11 | 11.81 ± 0.074 | 0.108 |
| CCM | 96.2 | 3.7 | 22.8 ± 0.5 | 5.47 ± 0.06 | 216.0 ± 0.4 | 27.34 ± 0.07 | 10.94 ± 0.04 | 0.102 |
| LCI | 94.1 | 5.9 | 27.8 ± 0.5 | 4.38 ± 0.08 | 218.1 ± 0.6 | 27.48 ± 0.08 | 11.55 ± 0.05 | 0.065 |
| MI | 96.3 | 3.7 | 26.0 ± 0.5 | 5.13 ± 0.07 | 219.6 ± 0.5 | 27.46 ± 0.08 | 11.16 ± 0.05 | 0.087 |
| NM | 94.4 | 5.6 | 22.2 ± 0.6 | 4.78 ± 0.09 | 216.9 ± 0.6 | 28.46 ± 0.09 | 11.84 ± 0.06 | 0.082 |
| OH | 95.5 | 4.5 | 17.6 ± 0.4 | 6.13 ± 0.07 | 215.1 ± 0.4 | 29.34 ± 0.07 | 11.61 ± 0.04 | 0.100 |
| PCM | 95.9 | 4.1 | 14.8 ± 0.5 | 5.65 ± 0.07 | 213.2 ± 0.4 | 29.33 ± 0.08 | 11.84 ± 0.05 | 0.122 |
| QI | 97.3 | 2.7 | 29.0 ± 0.4 | 5.02 ± 0.06 | 219.3 ± 0.39 | 26.18 ± 0.05 | 10.58 ± 0.04 | 0.082 |
| RB | 95.9 | 4.1 | 20.4 ± 0.4 | 5.16 ± 0.06 | 213.4 ± 0.38 | 28.69 ± 0.06 | 11.76 ± 0.04 | 0.085 |
| RCC | 94.4 | 5.6 | 22.5 ± 0.5 | 4.90 ± 0.09 | 216.3 ± 0.6 | 28.45 ± 0.09 | 11.77 ± 0.06 | 0.072 |
| SH | 95.6 | 4.4 | 22.0 ± 0.5 | 5.26 ± 0.08 | 218.4 ± 0.5 | 27.81 ± 0.07 | 11.28 ± 0.05 | 0.097 |
| SHS | 92.2 | 7.8 | 23.5 ± 0.7 | 5.61 ± 0.10 | 215.8 ± 0.6 | 28.63 ± 0.09 | 11.53 ± 0.06 | 0.089 |
| SS | 94.1 | 5.9 | 28.5 ± 0.5 | 4.27 ± 0.08 | 222.7 ± 0.6 | 26.71 ± 0.09 | 11.22 ± 0.06 | 0.068 |
| W (Full) | 99.4 | 0.6 | 19.0 ± 0.1 | 5.27 ± 0.02 | 212.3 ± 0.1 | 28.75 ± 0.02 | 11.74 ± 0.01 | 0.076 |
| W (Sub) |  |  | 18.7 ± 0.4 | 5.26 ± 0.06 | 212.3 ± 0.3 | 28.79 ± 0.04 | 11.76 ± 0.03 |  |
| WW (Full) | 99.8 | 0.2 | 16.5 ± 0.1 | 6.03 ± 0.02 | 211.6 ± 0.1 | 29.66 ± 0.02 | 11.81 ± 0.01 | 0.063 |
| WW (Sub) |  |  | 16.7 ± 0.3 | 6.00 ± 0.06 | 211.7 ± 0.3 | 29.69 ± 0.04 | 11.84 ± 0.03 |  |

| **Table S2**. VCR-LTER and ESL sites salinity (psu) composite harmonic elements where percent variance of the first harmonic ($pV_{1}$) and the second harmonic ($pV_{2}$) are in %, minimum, maximum, and amplitude ($A_{max}$) values are in psu and time of minima ($t_{min}$) and maxima ($t_{max}$) are in year day. | | | | | | | | |
| --- | --- | --- | --- | --- | --- | --- | --- | --- |
| Site | $pV_{1}$ | $pV_{2}$ | $t_{min}$ | Minimum Value | $t_{max}$ | Maximum Value | $A_{max}$ | $\chi_{v}^{2}$ |
| RBCM | 66.9 | 33.1 | 330.3 ± 2.4 | 29.05 ± 0.10 | 212.4 ± 2.8 | 31.98 ± 0.08 | 1.50 ± 0.07 | 0.901 |
| CCM | 28.0 | 72.0 | 44.7 ± 3.5 | 30.28 ± 0.04 | 303.4 ± 3.2 | 31.38 ± 0.04 | 0.55 ± 0.03 | 0.994 |
| LCI | 24.6 | 75.4 | 312.1 ± 2.8 | 30.64 ± 0.04 | 219.0 ± 3.4 | 31.57 ± 0.05 | 0.48 ± 0.03 | 0.985 |
| MI | 57.9 | 42.1 | 56.6 ± 4.9 | 30.88 ± 0.04 | 165.4 ± 4.8 | 31.60 ± 0.36 | 0.37 ± 0.03 | 1.021 |
| NM | 54.4 | 45.6 | 329.7 ± 4.7 | 30.80 ± 0.41 | 213.1 ± 5.8 | 31.56 ± 0.04 | 0.39 ± 0.03 | 1.024 |
| OH | 59.3 | 40.7 | 32.9 ± 3.3 | 28.83 ± 0.07 | 280.7 ± 4.7 | 30.24 ± 0.68 | 0.74 ± 0.06 | 1.007 |
| PCM | 85.2 | 14.8 | 61.5 ± 2.131 | 24.46 ± 0.11 | 202.4 ± 4.1 | 29.57 ± 0.12 | 2.55 ± 0.08 | 0.906 |
| QI | 60.6 | 39.4 | 48.7 ± 3.4 | 30.13 ± 0.05 | 293.5 ± 4.7 | 31.28 ± 0.05 | 0.59 ± 0.03 | 1.004 |
| RB | 95.7 | 4.3 | 53.8 ± 3.4 | 28.82 ± 0.06 | 277.4 ± 4.3 | 30.76 ± 0.06 | 0.97 ± 0.04 | 0.968 |
| RCC | 65.3 | 34.7 | 320.4 ± 4.2 | 30.75 ± 0.03 | 206.9 ± 5.8 | 31.47 ± 0.04 | 0.36 ± 0.03 | 1.030 |
| SH | 22.4 | 77.6 | 40.3 ± 3.6 | 30.76 ± 0.04 | 292.6 ± 4.9 | 31.53 ± 0.04 | 0.40 ± 0.03 | 1.019 |
| SHS | 44.9 | 55.1 | 308.3 ± 4.7 | 30.62 ± 0.05 | 61.55 ± 5.8 | 31.61 ± 0.05 | 0.51 ± 0.04 | 1.032 |
| SS | 17.2 | 82.8 | 303.7 ± 4.4 | 30.93 ± 0.03 | 202.9 ± 5.0 | 31.56 ± 0.03 | 0.32 ± 0.03 | 1.025 |
| W (Full) | 94.6 | 5.4 | 45.9 ± 0.5 | 28.74 ± 0.02 | 185.5 ± 0.92 | 31.61 ± 0.02 | 1.43 ± 0.01 | 0.793 |
| W (Sub) |  |  | 46.2 ± 1.2 | 28.71 ± 0.05 | 189.1 ± 2.2 | 31.7 ± 0.04 | 1.47 ± 0.03 |  |
| WW (Full) | 95.48 | 4.515 | 57.7 ± 0.2 | 25.18 ± 0.02 | 215.5 ± 0.8 | 32.50 ± 0.02 | 3.66 ± 0.01 | 0.357 |
| WW (Sub) |  |  | 57.2 ± 0.7 | 25.21 ± 0.05 | 218.1 ± 1.9 | 32.49 ± 0.03 | 3.64 ± 0.03 |  |

| **Table S3.** VCR-LTER and ESL sites dissolved oxygen (mg/L) composite harmonic elements where percent variance of the first harmonic ($pV_{1}$) and the second harmonic ($pV_{2}$) are in %, minimum, maximum, and amplitude ($A_{max}$) values are in mg/L and time of minima ($t_{min}$) and maxima ($t_{max}$) are in year day. | | | | | | | | |
| --- | --- | --- | --- | --- | --- | --- | --- | --- |
| Site | $pV_{1}$ | $pV_{2}$ | $t_{min}$ | Minimum Value | $t_{max}$ | Maximum Value | $A_{max}$ | $\chi_{v}^{2}$ |
| RBCM | 98.3 | 1.7 | 223.0 ± 4.3 | 6.15 ± 0.06 | 25.0 ± 1.2 | 11.15 ± 0.07 | 2.50 ± 0.05 | 0.574 |
| CCM | 97.9 | 2.1 | 213.4 ± 1.6 | 5.11 ± 0.04 | 32.5 ± 1.0 | 10.46 ± 0.04 | 2.68 ± 0.03 | 0.429 |
| LCI | 97.5 | 2.5 | 214.7 ± 3.3 | 6.45 ± 0.06 | 36.9 ± 1.4 | 11.74 ± 0.06 | 2.65 ± 0.04 | 0.435 |
| MI | 99.8 | 0.2 | 225.6 ± 3.2 | 6.26 ± 0.05 | 31.4 ± 1.1 | 10.84 ± 0.05 | 2.29 ± 0.03 | 0.516 |
| NM | 98.4 | 1.6 | 219.6 ± 2.7 | 5.66 ± 0.05 | 29.8 ± 1.0 | 11.66 ± 0.05 | 3.00 ± 0.03 | 0.331 |
| OH | 99.7 | 0.3 | 236.9 ± 3.4 | 6.23 ± 0.04 | 30.0 ± 1.5 | 10.93 ± 0.06 | 2.35 ± 0.04 | 0.550 |
| PCM | 99.8 | 0.2 | 208.9 ± 3.0 | 4.916 ± 0.04 | 26.3 ± 0.8 | 10.36 ± 0.04 | 2.72 ± 0.03 | 0.474 |
| QI | 98.8 | 1.2 | 209.3 ± 2.4 | 6.27 ± 0.04 | 31.5 ± 1.1 | 10.74 ± 0.04 | 2.24 ± 0.03 | 0.522 |
| RB | 99.0 | 1.0 | 206.2 ± 2.3 | 5.50 ± 0.04 | 27.0 ± 0.95 | 10.41 ± 0.04 | 2.46 ± 0.02 | 0.484 |
| RCC | 97.0 | 3.0 | 222.9 ± 2.3 | 5.85 ± 0.05 | 26.8 ± 1.1 | 11.21 ± 0.05 | 2.68 ± 0.04 | 0.368 |
| SH | 99.6 | 0.4 | 217.6 ± 3.4 | 6.07 ± 0.07 | 24.7 ± 1.1 | 11.05 ± 0.05 | 2.50 ± 0.03 | 0.512 |
| SHS | 95.5 | 4.5 | 225.1 ± 2.4 | 6.38 ± 0.05 | 21.1 ± 1.3 | 11.18 ± 0.05 | 2.40 ± 3.41 | 0.423 |
| SS | 96.3 | 3.7 | 217.9 ± 3.0 | 6.56 ± 0.06 | 35.5 ± 1.4 | 11.97 ± 0.06 | 2.71 ± 0.04 | 0.435 |
| W (Full) | 100.0 | 0.0 | 210.4 ± 0.3 | 4.94 ± 0.01 | 24.5 ± 0.1 | 10.55 ± 0.01 | 2.80 ± 0.004 | 0.110 |
| W (Sub) |  |  | 211.0 ± 0.3 | 4.92 ± 0.01 | 24.1 ± 0.20 | 10.53 ± 0.01 | 2.81 ± 0.01 |  |
| WW (Full) | 99.5 | 0.5 | 205.5 ± 0.3 | 5.18 ± 0.01 | 25.0 ± 0.1 | 10.38 ± 0.01 | 2.60 ± 0.004 | 0.120 |
| WW (Sub) |  |  | 205.8 ± 0.7 | 5.17 ± 0.01 | 24.8 ± 0.4 | 10.38 ± 0.02 | 2.61 ± 0.01 |  |

| **Table S4.** VCR-LTER and ESL sites chlorophyll-a converted to log_10_(Chl) (log_10_(ug/L)) composite harmonic elements where percent variance of the first harmonic ($pV_{1}$) and the second harmonic ($pV_{2}$) are in %, minimum, maximum, and amplitude ($A_{max}$) values are in log_10_(ug/L) and time of minima ($t_{min}$) and maxima ($t_{max}$) are in year day. | | | | | | | | |
| --- | --- | --- | --- | --- | --- | --- | --- | --- |
| Site | $pV_{1}$ | $pV_{2}$ | $t_{min}$ | Minimum Value | $t_{max}$ | Maximum Value | $A_{max}$ | $\chi_{v}^{2}$ |
| RBCM | 57.6 | 42.4 | 333.2 ± 2.5 | 0.26 ± 0.02 | 221.9 ± 1.2 | 1.07 ± 0.02 | 0.40 ± 0.01 | 0.627 |
| CCM | 52.3 | 47.7 | 321.5 ± 1.6 | 0.20 ± 0.01 | 213.1 ± 1.2 | 0.66 ± 0.01 | 0.23 ± 0.01 | 0.844 |
| LCI | 66.1 | 33.9 | 114.8 ± 3.2 | 0.43 ± 0.01 | 237.7 ± 3.0 | 0.84 ± 0.01 | 0.20 ± 0.01 | 0.888 |
| MI | 55.8 | 44.2 | 116.3 ± 2.2 | 0.34 ± 0.01 | 228.8 ± 2.1 | 0.81 ± 0.01 | 0.23 ± 0.01 | 0.884 |
| NM | 25.3 | 74.7 | 126.9 ± 2.5 | 0.34 ± 0.01 | 227.5 ± 2.4 | 0.68 ± 0.01 | 0.18 ± 0.01 | 0.947 |
| OH | 82.3 | 17.7 | 341.5 ± 1.6 | 0.10 ± 0.01 | 205.4 ± 0.98 | 0.98 ± 0.01 | 0.45 ± 0.01 | 0.628 |
| PCM | 62.0 | 38.0 | 326.9 ± 0.9 | 0.11 ± 0.01 | 212.7 ± 0.8 | 1.1 ± 0.01 | 0.49 ± 0.01 | 0.637 |
| QI | 40.4 | 59.6 | 113.5 ± 1.6 | 0.36 ± 0.01 | 218.9 ± 1.4 | 0.75 ± 0.01 | 0.19 ± 0.01 | 0.919 |
| RB | 45.5 | 54.5 | 317.6 ± 2.4 | 0.23 ± 0.01 | 210.4 ± 2.1 | 0.61 ± 0.01 | 0.19 ± 0.01 | 0.956 |
| RCC | 49.6 | 50.4 | 117.5 ± 2.1 | 0.25 ± 0.02 | 226.1 ± 2.1 | 0.81 ± 0.02 | 0.28 ± 0.01 | 0.839 |
| SH | 41.1 | 58.9 | 319.2 ± 1.1 | 0.14 ± 0.01 | 216.5 ± 0.9 | 0.83 ± 0.01 | 0.34 ± 0.01 | 0.725 |
| SHS | 53.2 | 46.8 | 324.6 ± 2.2 | 0.47 ± 0.01 | 212.7 ± 1.4 | 0.99 ± 0.01 | 0.26 ± 0.01 | 0.715 |
| SS | 46.6 | 53.4 | 100.7 ± 2.8 | 0.51 ± 0.01 | 332.4 ± 4.5 | 0.83 ± 0.02 | 0.16 ± 0.01 | 0.951 |
| W (Full) | 57.3 | 42.7 | 317.8 ± 0.14 | -.03 ± 0.002 | 207.3 ± 0.2 | 1.05 ± 0.003 | 0.54 ± 0.002 | 0.430 |
| W (Sub) |  |  | 318.8 ± 0.49 | -.04 ± 0.01 | 207.3 ± 0.3 | 1.05 ± 0.01 | 0.55 ± 0.01 |  |
| WW (Full) | 38.1 | 61.9 | 325.8 ± 0.2 | 0.17 ± 0.003 | 220.4 ± 0.5 | 1.09 ± 0.003 | 0.46 ± 0.002 | 0.585 |
| WW (Sub) |  |  | 325.6 ± 0.5 | 0.17 ± 0.003 | 220.4 ± 0.5 | 1.09 ± 0.003 | 0.46 ± 0.003 |  |

| **Table S5.** VCR-LTER and ESL sites AOU (mg/L) composite harmonic elements where percent variance of the first harmonic ($pV_{1}$) and the second harmonic ($pV_{2}$) are in %, minimum, maximum, and amplitude ($A_{max}$) values are in mg/L and time of minima ($t_{min}$) and maxima ($t_{max}$) are in year day. | | | | | | | | |
| --- | --- | --- | --- | --- | --- | --- | --- | --- |
| Site | $pV_{1}$ | $pV_{2}$ | $t_{min}$ | Minimum Value | $t_{max}$ | Maximum Value | $A_{max}$ | $\chi_{v}^{2}$ |
| RBCM | 85.2 | 14.8 | 37.3 ± 2.5 | -1.12 ± 0.05 | 277.9 ± 4.5 | 0.69 ± 0.05 | 0.91 ± 0.04 | 0.957 |
| CCM | 94.6 | 5.4 | 40.9 ± 3.2 | -0.35 ± 0.04 | 210.6 ± 4.6 | 1.63 ± 0.05 | 0.99 ± 0.04 | 0.821 |
| LCI | 98.6 | 1.4 | 49.7 ± 3.9 | -1.17 ± 0.06 | 196.0 ± 5.4 | 0.25 ± 0.06 | 0.71 ± 0.04 | 0.988 |
| MI | 90.0 | 10.0 | 37.4 ± 2.9 | -0.69 ± 0.04 | 171.7 ± 4.6 | 0.58 ± 0.04 | 0.64 ± 0.03 | 0.977 |
| NM | 87.0 | 13.0 | 46.3 ± 4.0 | -0.66 ± 0.05 | 225.3 ± 3.5 | 1.08 ± 0.05 | 0.85 ± 0.04 | 0.851 |
| OH | 78.1 | 21.9 | 32.8 ± 2.6 | -0.79 ± 0.04 | 276.1 ± 3.3 | 0.63 ± 0.04 | 0.72 ± 0.03 | 0.971 |
| PCM | 92.2 | 7.8 | 37.1 ± 1.9 | 0.07 ± 0.04 | 272.1 ± 4.1 | 1.77 ± 0.03 | 0.86 ± 0.03 | 0.914 |
| QI | 85.5 | 14.5 | 44.2 ± 3.0 | -0.45 ± 0.04 | 175.3 ± 3.5 | 0.68 ± 0.03 | 0.57 ± 0.03 | 0.954 |
| RB | 99.8 | 0.2 | 27.0 ± 3.5 | -0.29 ± 0.50 | 221.7 ± 5.1 | 1.17 ± 0.03 | 0.73 ± 3.0 | 0.902 |
| RCC | 88.5 | 11.5 | 46.3 ± 4.4 | -0.66 ± 0.07 | 242.0 ± 3.5 | 0.87 ± 0.05 | 0.75 ± 0.04 | 0.911 |
| SH | 97.5 | 2.5 | 24.7 ± 2.6 | -1.00 ± 0.05 | 250.5 ± 3.8 | 0.69 ± 0.04 | 0.85 ± 0.03 | 0.904 |
| SHS | 96.7 | 3.3 | 47.5 ± 5.0 | -0.70 ± 0.06 | 246.8 ± 4.7 | 0.13 ± 0.06 | 0.43 ± 0.04 | 1.04 |
| SS | 92.6 | 7.4 | 55.7 ± 4.6 | -1.08 ± 0.06 | 219.9 ± 4.9 | 0.04 ± 0.07 | 0.56 ± 0.04 | 1.01 |
| W (Full) | 98.5 | 1.5 | 37.8 ± 0.3 | -0.10 ± 0.004 | 200.8 ± 0.4 | 1.54 ± 0.004 | 0.82 ± 0.002 | 0.410 |
| W (Sub) |  |  | 37.5 ± 0.8 | -0.10 ± 0.01 | 201.7 ± 1.2 | 1.54 ± 0.01 | 0.82 ± 0.01 |  |
| WW (Full) | 97.5 | 2.5 | 34.0 ± 0.6 | 0.07 ± 0.01 | 190.2 ± 0.6 | 1.23 ± 0.01 | 0.58 ± 0.003 | 0.633 |
| WW (Sub) |  |  | 34.0 ± 1.4 | 0.07 ± 0.01 | 194.2 ± 3.0 | 1.23 ± 0.02 | 0.58 ± 0.01 |  |

| **Table S6.** Average RMSE, nRMSE and NSE values from differences between the sub-sampling at the rate of low-frequency VCR-LTER sites versus the full high-frequency harmonic fit at ESL sites | | | | | |
| --- | --- | --- | --- | --- | --- |
| Site | Parameter | Number of Subsamples | Average RMSE | Average nRMSE | Average NSE |
| W | Temperature | 202 | 0.780 ± 0.019 °C | 0.345 ± 0.008 | 0.991 ± 0.0005 |
|  | Salinity | 190 | 0.710 ± 0.017 psu | 0.402 ± 0.010 | 0.865 ± 0.007 |
|  | Dissolved Oxygen | 175 | 0.469 ± 0.012 mg/L | 0.707 ± 0.019 | 0.943 ± 0.003 |
|  | Log_10_(Chl) | 164 | 0.330 ± 0.009 log_10_(ug/L) | 1.217 ± 0.034 | 0.296 ± 0.041 |
|  | AOU | 164 | 0.406 ± 0/009 mg/L | 0.789 ± 0.018 | 0.659 ± 0.018 |
| WW | Temperature | 202 | 0.761 ± 0.0183 °C | 0.360 ± 0.009 | 0.992 ± 0.0004 |
|  | Salinity | 190 | 0.745 ± 0.019 psu | 0.388 ± 0.010 | 0.943 ± 0.003 |
|  | Dissolved Oxygen | 175 | 0.464 ± 0.012 mg/L | 0.692 ± 0.017 | 0.940 ± 0.003 |
|  | Log_10_Chl) | 164 | 0.215 ± 0.003 log_10_(ug/L) | 0.695 ± 0.011 | 0.713 ± 0.011 |
|  | AOU | 164 | 0.437 ± 0.011 mg/L | 0.965 ± 0.025 | 0.532 ± 0.027 |

| **Table S7.** Long-term changes in water quality parameters at VCR-LTER sites measured at a low-frequency, where statistically significant (p < .05) values are bolded. | | | |
| --- | --- | --- | --- |
| Parameter | Site | Rate of Change | P-Value |
| Temperature | RBCM | 0.091 ± 0.090 °C/year | **0.049** |
|  | CCM | 0.023 ± 0.024 °C/year | 0.330 |
|  | LCI | 0.033 ± 0.072 °C/year | 0.374 |
|  | MI | -0.016 ± 0.063 °C/year | 0.619 |
|  | NM | 0.047 ± 0.084°C/year | 0.257 |
|  | OH | 0.025 ± 0.049 °C/year | 0.921 |
|  | PCM | 0.068 ± 0.051 °C/year | **0.001** |
|  | QI | 0.004 ± 0.041 °C/year | 0.847 |
|  | RB | 0.053 ± 0.043 °C/year | **0.017** |
|  | RCC | 0.055 ± 0.078 °C/year | 0.168 |
|  | SH | 0.012 ± 0.061 °C/year | 0.701 |
|  | SHS | 0.081 ± 0.098 °C/year | 0.112 |
|  | SS | 0.050 ± 0.077 °C/year | 0.208 |
| Salinity | RBCM | 0.073 ± 0.138 psu/year | 0.124 |
|  | CCM | 0.067 ± 0.036 psu/year | **0.003** |
|  | LCI | 0.044 ± 0.052 psu/year | 0.104 |
|  | MI | 0.025 ± 0.045 psu/year | 0.285 |
|  | NM | 0.045 ± 0.060 psu/year | 0.145 |
|  | OH | 0.080 ± 0.053 psu/year | **0.004** |
|  | PCM | 0.099 ± 0.093 psu/year | **0.038** |
|  | QI | 0.068 ± 0.038 psu/year | **0.001** |
|  | RB | 0.075 ± 0.057 psu/year | **0.011** |
|  | RCC | 0.042 ± 0.051 psu/year | 0.115 |
|  | SH | 0.025 ± 0.038 psu/year | 0.200 |
|  | SHS | 0.069 ± 0.076 psu/year | 0.080 |
|  | SS | 0.042 ± 0.047 psu/year | 0.080 |
| Log10(Chl) | RBCM | 0.015 ± 0.014 log_10_(ug/L)/year | **0.031** |
|  | CCM | 0.009 ± 0.008 log_10_(ug/L)/year | **0.023** |
|  | LCI | 0.014 ± 0.010 log_10_(ug/L)/year | 0.079 |
|  | MI | 0.024 ± 0.009 log_10_(ug/L)/year | **4.83 x 10^-07^** |
|  | NM | 0.014 ± 0.010 log_10_(ug/L)/year | 0.094 |
|  | OH | 0.0002 ± 0.007 log_10_(ug/L)/year | 0.969 |
|  | PCM | 0.013 ± 0.010 log_10_(ug/L)/year | **0.009** |
|  | QI | 0.011 ± 0.011 log_10_(ug/L)/year | **0.048** |
|  | RB | 0.014 ± 0.010 log_10_(ug/L)/year | **0.005** |
|  | RCC | 0.027 ± 0.017 log_10_(ug/L)/year | **0.004** |
|  | SH | 0.025 ± 0.009 log_10_(ug/L)/year | **1.08 x 10^-07^** |
|  | SHS | 0.011 ± 0.010 log_10_(ug/L)/year | 0.167 |
|  | SS | 0.003 ± 0.012 log_10_(ug/L)/year | 0.751 |
| AOU | RBCM | -0.057 ± 0.055 mg/L/year | **0.041** |
|  | CCM | 0.002 ± 0.036 mg/L/year | 0.903 |
|  | LCI | 0.036 ± 0.087 mg/L/year | 0.418 |
|  | MI | -0.042 ± 0.051 mg/L/year | 0.108 |
|  | NM | -0.007 ± 0.079 mg/L/year | 0.862 |
|  | OH | 0.191 ± 0.035 mg/L/year | 0.290 |
|  | PCM | -0.035 ± 0.036 mg/L/year | 0.058 |
|  | QI | -0.010 ± 0.037 mg/L/year | 0.611 |
|  | RB | -0.017 ± 0.030 mg/L/year | 0.262 |
|  | RCC | -0.057 ± 0.055 mg/L/year | 0.909 |
|  | SH | -0.044 ± 0.043 mg/L/year | **0.047** |
|  | SHS | -0.011 ± 0.078 mg/L/year | 0.792 |
|  | SS | 0.049 ± 0.071 mg/L/year | 0.180 |

| **Table S8**: Two-tailed unequal variance t-tests between mainland and ocean-inlet and mid-lagoon sites’ harmonic elements where statistically significant values (p<0.05) are bolded | | | |
| --- | --- | --- | --- |
| Variable |  | t Critical two-tail | P-Value |
| Temperature | Date of Maximum | 2.179 | **0.0005** |
|  | Maximum Value | 2.160 | **0.0005** |
|  | Seasonal Amplitude | 2.306 | **0.007** |
| Salinity | Date of Maximum | 2.228 | 0.804 |
|  | Maximum Value | 2.447 | 0.416 |
|  | Seasonal Amplitude | 2.447 | **0.037** |
| Dissolved Oxygen | Date of Maximum | 2.306 | 0.759 |
|  | Maximum Value | 2.179 | 0.075 |
|  | Seasonal Amplitude | 2.179 | 0.964 |
| Log10(Chl) | Date of Maximum | 2.306 | 0.229 |
|  | Maximum Value | 2.262 | **0.034** |
|  | Seasonal Amplitude | 2.306 | **0.009** |
| AOU | Date of Maximum | 2.179 | 0.112 |
|  | Maximum Value | 2.160 | 0.066 |
|  | Seasonal Amplitude | 2.201 | 0.404 |

| **Table S9.** Differences between earlier and later time periods of long-term low-frequency VCR-LTER sites. Bolded variables indicate statistically significant changes between the two time periods. | | | | |
| --- | --- | --- | --- | --- |
| Site | Parameter | Difference in Min/Max Date | Difference in Min/Max Value | Difference in Seasonal Amplitude |
| RBCM | **Temperature** | -1.8 day | 0.865 °C | 1.370 °C |
|  | Salinity | 19.0 days | 0.738 psu | 0.089 psu |
|  | DO | 29.6 days | 0.754 mg/L | 0.112 mg/L |
|  | **Log10(Chl)** | 22.2 days | 0.005 log_10_(ug/L) | -0.101 log_10_(ug/L) |
|  | **AOU** | 27.9 days | -1.051 mg/L | -0.038 mg/L |
| CCM | Temperature | 4.4 days | 0.203 °C | 0.426 °C |
|  | **Salinity** | -72.3 days | 0.815 psu | -0.213 psu |
|  | DO | 15.2 days | -0.692 mg/L | 0.765 mg/L |
|  | **Log10(Chl)** | 6.9 days | 0.166 log_10_(ug/L) | -0.067 log_10_(ug/L) |
|  | AOU | 18.8 days | 0.288 mg/L | 0.441 mg/L |
| LCI | Temperature | -5.6 days | -0.338 °C | 0.196 °C |
|  | Salinity | 6.6 days | 0.583 psu | 0.134 psu |
|  | DO | -26.1 days | -0.039 mg/L | 0.025 mg/L |
|  | Log10(Chl) | -17.9 days | 0.143 log_10_(ug/L) | 0.009 log_10_(ug/L) |
|  | AOU | -54.0 days | 0.299 mg/L | 0.384 mg/L |
| MI | Temperature | -4.5 days | 0.160 °C | 0.628 °C |
|  | Salinity | -110.8 days | 0.778 psu | 0.228 psu |
|  | DO | -30.5 days | 0.673 mg/L | 0.161 mg/L |
|  | **Log10(Chl)** | 11.9 days | 0.321 log_10_(ug/L) | -0.041 log_10_(ug/L) |
|  | AOU | -42.6 days | -0.518 mg/L | 0.090 mg/L |
| NM | Temperature | -5.9 days | 0.3992 °C | 0.742 °C |
|  | Salinity | -116.5 days | 0.861 psu | 0.421 psu |
|  | DO | -39.9 days | 0.889 mg/L | -0.559 mg/L |
|  | Log10(Chl) | 22.6 days | 0.124 log_10_(ug/L) | -0.066 log_10_(ug/L) |
|  | AOU | 41.0 days | -0.814 mg/L | -0.528 mg/L |
| OH | Temperature | 1.5 days | -0.303 °C | 0.767 °C |
|  | **Salinity** | 103.4 days | 1.966 psu | 0.498 psu |
|  | DO | -1.5 days | -0.856 mg/L | 0.812 mg/L |
|  | Log10(Chl) | 7.6 days | 0.020 log_10_(ug/L) | -0.028 log_10_(ug/L) |
|  | AOU | -16.2 days | 0.693 mg/L | 0.351 mg/L |
| PCM | **Temperature** | -2.6 days | 0.919 °C | 0.333 °C |
|  | **Salinity** | 43.8 days | 3.416 psu | 0.939 psu |
|  | DO | -25.3 days | 0.699 mg/L | 0.020 mg/L |
|  | Log10(Chl) | 11.4 days | 0.348 log_10_(ug/L) | 0.002 log_10_(ug/L) |
|  | AOU | -119.2 days | -0.515 mg/L | 0.103 mg/L |
| QI | Temperature | 9.5 days | -0.475 °C | -0.112 °C |
|  | **Salinity** | -70.2 days | 0.602 psu | -0.369 psu |
|  | DO | 15.5 days | 0.356 mg/L | 0.354 mg/L |
|  | **Log10(Chl)** | -16.7 days | 0.254 log_10_(ug/L) | -0.012 log_10_(ug/L) |
|  | AOU | -5.3 days | -0.156 mg/L | 0.467 mg/L |
| RB | **Temperature** | 4.4 days | 0.746 °C | 0.564 °C |
|  | **Salinity** | -72.8 days | 1.841 psu | 0.019 psu |
|  | DO | 32.4 days | 0.161 mg/L | 0.443 mg/L |
|  | **Log10(Chl)** | 40.9 days | 0.279 log_10_(ug/L) | -0.012 log_10_(ug/L) |
|  | AOU | -70.3 days | -0.291 mg/L | 0.170 mg/L |
| RCC | Temperature | -4.6 days | 0.413 °C | 0.428 °C |
|  | Salinity | 76.2 days | 0.595 psu | 0.215 psu |
|  | DO | -42.4 days | 0.641 mg/L | -0.001 mg/L |
|  | **Log10(Chl)** | -2.7 days | 0.222 log_10_(ug/L) | -0.135 log_10_(ug/L) |
|  | AOU | 42.8 days | -0.437 mg/L | -0.282 mg/L |
| SH | Temperature | -5.8 days | 0.375 °C | 0.941 °C |
|  | Salinity | -243.8 days | 0.904 psu | 0.267 psu |
|  | DO | -10.9 days | 0.330 mg/L | 0.194 mg/L |
|  | **Log10(Chl)** | -2.7 days | 0.280 log_10_(ug/L) | -0.119 log_10_(ug/L) |
|  | **AOU** | -99.0 days | -0.568 mg/L | -0.184 mg/L |
| SHS | Temperature | -3.8 days | 0.764 °C | 0.870 °C |
|  | Salinity | -19.3 days | 0.922 psu | 0.266 psu |
|  | DO | 8.7 days | 0.595 mg/L | -0.310 mg/L |
|  | Log10(Chl) | -2.0 days | -0.011 log_10_(ug/L) | -0.109 log_10_(ug/L) |
|  | AOU | -142.2 days | -0.496 mg/L | -0.462 mg/L |
| SS | Temperature | 0.898 days | -0.041 °C | 0.108 °C |
|  | Salinity | -164.7 days | 0.657 psu | 0.054 psu |
|  | DO | -26.4 days | -0.013 mg/L | -0.447 mg/L |
|  | Log10(Chl) | 116.5 days | 0.188 log_10_(ug/L) | 0.077 log_10_(ug/L) |
|  | AOU | -60.1 days | 0.672 mg/L | 0.323 mg/L |

| **Table S10**: Two-tailed unequal variance t-tests between decade segments’ harmonic elements where statistically significant values (p<0.05) are bolded. | | | | |
| --- | --- | --- | --- | --- |
|  | Variable |  | t Critical two-tail | P-Value |
| All Sites | Temperature | Date of Maximum | 2.080 | 0.505 |
|  |  | Maximum Value | 2.074 | 0.551 |
|  |  | Seasonal Amplitude | 2.086 | **0.005** |
|  | Salinity | Date of Maximum | 2.069 | 0.118 |
|  |  | Maximum Value | 2.145 | **0.0002** |
|  |  | Seasonal Amplitude | 2.074 | 0.516 |
|  | Dissolved Oxygen | Date of Maximum | 2.101 | 0.308 |
|  |  | Maximum Value | 2.064 | 0.268 |
|  |  | Seasonal Amplitude | 2.080 | 0.340 |
|  | Log10(Chl) | Date of Maximum | 2.145 | 0.173 |
|  |  | Maximum Value | 2.064 | **0.010** |
|  |  | Seasonal Amplitude | 2.064 | 0.290 |
|  | AOU | Date of Maximum | 2.101 | 0.093 |
|  |  | Maximum Value | 2.064 | 0.322 |
|  |  | Seasonal Amplitude | 2.069 | 0.497 |
| Mainland | Temperature | Date of Maximum | 2.365 | 0.696 |
|  |  | Maximum Value | 2.306 | 0.280 |
|  |  | Seasonal Amplitude | 2.365 | **0.001** |
|  | Salinity | Date of Maximum | 2.278 | 0.357 |
|  |  | Maximum Value | 2.776 | **0.024** |
|  |  | Seasonal Amplitude | 2.365 | 0.585 |
|  | Dissolved Oxygen | Date of Maximum | 2.447 | 0.930 |
|  |  | Maximum Value | 2.365 | 0.476 |
|  |  | Seasonal Amplitude | 2.365 | 0.094 |
|  | Log10(Chl) | Date of Maximum | 2.306 | **0.044** |
|  |  | Maximum Value | 2.306 | 0.225 |
|  |  | Seasonal Amplitude | 2.306 | 0.486 |
|  | AOU | Date of Maximum | 2.571 | 0.493 |
|  |  | Maximum Value | 2.306 | 0.374 |
|  |  | Seasonal Amplitude | 2.365 | 0.510 |
| Ocean Inlet and Mid-lagoon | Temperature | Date of Maximum | 2.178 | 0.721 |
|  |  | Maximum Value | 2.201 | 0.762 |
|  |  | Seasonal Amplitude | 2.228 | 0.149 |
|  | Salinity | Date of Maximum | 2.179 | **0.025** |
|  |  | Maximum Value | 2.179 | **0.0001** |
|  |  | Seasonal Amplitude | 2.201 | 0.712 |
|  | Dissolved Oxygen | Date of Maximum | 2.201 | 0.175 |
|  |  | Maximum Value | 2.160 | 0.278 |
|  |  | Seasonal Amplitude | 2.160 | 0.987 |
|  | Log10(Chl) | Date of Maximum | 2.306 | 0.330 |
|  |  | Maximum Value | 2.145 | **0.007** |
|  |  | Seasonal Amplitude | 2.201 | 0.153 |
|  | AOU | Date of Maximum | 2.228 | **0.038** |
|  |  | Maximum Value | 2.145 | 0.440 |
|  |  | Seasonal Amplitude | 2.179 | 0.837 |

| **Table S11**. Duration of quarterly sampling needed for the sub-sampled low-frequency and high-frequency confidence intervals to overlap for harmonic elements for sites W and WW. | | | |
| --- | --- | --- | --- |
| Site | Variable | Composite Harmonic Element | Years to Reach Confidence Intervals |
| W | Temperature | Date of Maximum | 19 |
|  |  | Maximum | 20 |
|  |  | Seasonal Amplitude | 18 |
|  |  | *Average* | 19.0 ± 1.3 |
|  | Salinity | Date of Maximum | 27 |
|  |  | Maximum | 12 |
|  |  | Seasonal Amplitude | 31 |
|  |  | *Average* | 23.3 ± 11.3 |
|  | Dissolved Oxygen | Date of Minimum | 11 |
|  |  | Minimum | 17 |
|  |  | Seasonal Amplitude | 12 |
|  |  | *Average* | 13.3 ± 3.6 |
|  | Log10(Chl) | Date of Maximum | 3** |
|  |  | Maximum | 18 |
|  |  | Seasonal Amplitude | 5* |
|  |  | *Average* | 11.5 ± 10.8* |
|  | AOU | Date of Maximum | 11* |
|  |  | Maximum | 15 |
|  |  | Seasonal Amplitude | 31 |
|  |  | *Average* | 19 ± 12.0* |
| WW | Temperature | Date of Maximum | 5 |
|  |  | Maximum | 7 |
|  |  | Seasonal Amplitude | 18 |
|  |  | *Average* | 10.0 ± 7.9 |
|  | Salinity | Date of Maximum | 10 |
|  |  | Maximum | 14 |
|  |  | Seasonal Amplitude | 11 |
|  |  | *Average* | 11.7 ± 2.4 |
|  | Dissolved Oxygen | Date of Minimum | 18 |
|  |  | Minimum | 12 |
|  |  | Seasonal Amplitude | 21 |
|  |  | *Average* | 17.0 ± 5.2 |
|  | Log10(Chl) | Date of Maximum | 9 |
|  |  | Maximum | 15 |
|  |  | Seasonal Amplitude | 15 |
|  |  | *Average* | 13.0 ± 2.9 |
|  | AOU | Date of Maximum | 28 |
|  |  | Maximum | 17 |
|  |  | Seasonal Amplitude | 24 |
|  |  | *Average* | 23.0 ± 6.3 |
| Where * indicates a value that only crosses the harmonic element confidence intervals for a singular point, and should be taken with caution and ** is the date with the closest point that does not cross, and is not included in the average. | | | |

| **Table S12**. Duration of quarterly sampling needed for the sub-sampled low-frequency and high-frequency confidence intervals to overlap for harmonic model parameters for sites W and WW. | | | |
| --- | --- | --- | --- |
| Site | Variable | Harmonic Element | Years to Reach Confidence Intervals |
| W | Temperature | ȳ | 12 |
|  |  | a1 | 20 |
|  |  | b1 | 18 |
|  |  | a2 | 22 |
|  |  | b2 | 19 |
|  |  | *Average* | 18.2 ± 3.3 |
|  | Salinity | ȳ | 17 |
|  |  | a1 | 22 |
|  |  | b1 | 16 |
|  |  | a2 | 31* |
|  |  | b2 | 9* |
|  |  | *Average* | 19.0 ± 7.1* |
|  | DO | ȳ | 4 |
|  |  | a1 | 6 |
|  |  | b1 | 34 |
|  |  | a2 | 5 |
|  |  | b2 | 8 |
|  |  | *Average* | 11.4 ± 11.1 |
|  | Log10(Chl) | ȳ | 9** |
|  |  | a1 | 30 |
|  |  | b1 | 3** |
|  |  | a2 | 4 |
|  |  | b2 | 3** |
|  |  | *Average* | 17.0 ± 25.5 |
|  | AOU | ȳ | 18 |
|  |  | a1 | 20 |
|  |  | b1 | 10 |
|  |  | a2 | 14* |
|  |  | b2 | 2* |
|  |  | *Average* | 12.8 ± 6.3* |
| WW | Temperature | ȳ | 13 |
|  |  | a1 | 11 |
|  |  | b1 | 38 |
|  |  | a2 | 18 |
|  |  | b2 | 11 |
|  |  | *Average* | 18.2 ± 10.0 |
|  | Salinity | ȳ | 16 |
|  |  | a1 | 18 |
|  |  | b1 | 17 |
|  |  | a2 | 26 |
|  |  | b2 | 4 |
|  |  | *Average* | 14.2 ± 5.1 |
|  | DO | ȳ | 8 |
|  |  | a1 | 21 |
|  |  | b1 | 9 |
|  |  | a2 | 16 |
|  |  | b2 | 36 |
|  |  | *Average* | 18.0 ± 10.0 |
|  | Log10(Chl) | ȳ | 32 |
|  |  | a1 | 20 |
|  |  | b1 | 20 |
|  |  | a2 | 15 |
|  |  | b2 | 26 |
|  |  | *Average* | 22.6 ± 5.7 |
|  | AOU | ȳ | 14 |
|  |  | a1 | 12 |
|  |  | b1 | 18 |
|  |  | a2 | 16 |
|  |  | b2 | 6 |
|  |  | *Average* | 13.2 ± 4.0 |
| Where a star (*) indicates a year value where only one point reaches within the confidence intervals, and ** where no value reaches within the confidence interval, but this is the closest year. Doubled starred values (**) are not included in the average calculations. | | | |

| **Table S13.** nRMSE values between simulated low-frequency subsampled and full high-frequency data at sites W and WW. | | | | |
| --- | --- | --- | --- | --- |
| Site | Parameter | nRMSE at 5 years | nRMSE at 15 years | nRMSE at 50 years |
| W | Temperature | 0.457 | 0.231 | 0.129 |
|  | Salinity | 0.679 | 0.421 | 0.169 |
|  | Dissolved Oxygen | 1.114 | 0.468 | 0.266 |
|  | Log_10_(Chl) | 2.112 | 1.438 | 0.757 |
|  | AOU | 0.884 | 0.651 | 0.291 |
| WW | Temperature | 0.464 | 0.238 | 0.081 |
|  | Salinity | 0.639 | 0.247 | 0.105 |
|  | Dissolved Oxygen | 0.974 | 0.499 | 0.223 |
|  | Log_10_Chl) | 1.884 | 0.542 | 0.292 |
|  | AOU | 1.099 | 0.542 | 0.333 |

| **Table S14.** Average nSD values of simulated low-frequency and full high-frequency data for harmonic elements of amplitudes and dates of maximum/minimums. | | | | |
| --- | --- | --- | --- | --- |
| Site | Parameter | Average nSD at 5 years | Average nSD at 15 years | Average nSD at 50 years |
| W | Temperature | 5.612 | 3.216 | 1.708 |
|  | Salinity | 7.316 | 3.516 | 1.762 |
|  | Dissolved Oxygen | 5.394 | 3.096 | 1.668 |
|  | Log_10_(Chl) | 7.313 | 3.022 | 1.801 |
|  | AOU | 5.440 | 3.222 | 1.781 |
| WW | Temperature | 5.919 | 3.376 | 1.718 |
|  | Salinity | 5.453 | 3.258 | 1.795 |
|  | Dissolved Oxygen | 5.586 | 3.324 | 1.637 |
|  | Log_10_Chl) | 6.820 | 3.188 | 1.684 |
|  | AOU | 5.578 | 3.195 | 1.630 |
